# Promoter methylation, transcription, and retrotransposition of LINE-1 in colorectal adenomas and adenocarcinomas

**DOI:** 10.1186/s12935-020-01511-5

**Published:** 2020-09-01

**Authors:** Milad Shademan, Khadijeh Zare, Morteza Zahedi, Hooman Mosannen Mozaffari, Hadi Bagheri Hosseini, Kamran Ghaffarzadegan, Ladan Goshayeshi, Hesam Dehghani

**Affiliations:** 1 Graduate Program in Physiology, Department of Basic Sciences, Faculty of Veterinary Medicine, Ferdowsi University of Mashhad, Mashhad, Iran; 2 Stem Cell Biology and Regenerative Medicine Research Group, Research Institute of Biotechnology, Ferdowsi University of Mashhad, Azadi Square, Mashhad, 91779-48974 Iran; 3 Department of Gastroenterology and Hepatology, Faculty of Medicine, Mashhad University of Medical Sciences, Mashhad, Iran; 4 Gastroenterology and Hepatology Research Center, Mashhad University of Medical Sciences, Mashhad, Iran; 5 Pathology Department, Education and Research Department, Razavi Hospital, Mashhad, Iran; 6 Surgical Oncology Research Center, Mashhad University of Medical Sciences, Mashhad, Iran; 7 Division of Biotechnology, Faculty of Veterinary Medicine, Ferdowsi University of Mashhad, Mashhad, Iran; 8 Department of Basic Sciences, Faculty of Veterinary Medicine, Ferdowsi University of Mashhad, Mashhad, Iran

**Keywords:** LINE-1, Colorectal adenoma, Colorectal adenocarcinoma, Methylation, Retrotransposition, Insertion

## Abstract

**Background:**

The methylation of the CpG islands of the LINE-1 promoter is a tight control mechanism on the function of mobile elements. However, simultaneous quantification of promoter methylation and transcription of LINE-1 has not been performed in progressive stages of colorectal cancer. In addition, the insertion of mobile elements in the genome of advanced adenoma stage, a precancerous stage before colorectal carcinoma has not been emphasized. In this study, we quantify promoter methylation and transcripts of LINE-1 in three stages of colorectal non-advanced adenoma, advanced adenoma, and adenocarcinoma. In addition, we analyze the insertion of LINE-1, Alu, and SVA elements in the genome of patient tumors with colorectal advanced adenomas.

**Methods:**

LINE-1 hypomethylation status was evaluated by absolute quantitative analysis of methylated alleles (AQAMA) assay. To quantify the level of transcripts for LINE-1, quantitative RT-PCR was performed. To find mobile element insertions, the advanced adenoma tissue samples were subjected to whole genome sequencing and MELT analysis.

**Results:**

We found that the LINE-1 promoter methylation in advanced adenoma and adenocarcinoma was significantly lower than that in non-advanced adenomas. Accordingly, the copy number of LINE-1 transcripts in advanced adenoma was significantly higher than that in non-advanced adenomas, and in adenocarcinomas was significantly higher than that in the advanced adenomas. Whole-genome sequencing analysis of colorectal advanced adenomas revealed that at this stage polymorphic insertions of LINE-1, Alu, and SVA comprise approximately 16%, 51%, and 74% of total insertions, respectively.

**Conclusions:**

Our correlative analysis showing a decreased methylation of LINE-1 promoter accompanied by the higher level of LINE-1 transcription, and polymorphic genomic insertions in advanced adenoma, suggests that the early and advanced polyp stages may host very important pathogenic processes concluding to cancer.

## Background

Mobile pieces of DNA called transposable elements occupy more than half of the human genome [1, 2]. Insertion of mobile elements into the genome of somatic cells and their movement may lead to cancers. DNA transposons and retrotransposons are the two major classes of mobile elements. Retrotransposons comprise two main groups of human endogenous retroviruses and poly(A) retrotransposons [2]. In the latter group, LINE-1, Alu, and SVA (SINE-R-VNTR-Alus) make up approximately one-third of the human mobile DNA. One of the most active DNA retrotransposons in mammals is LINE-1, which contains 17% of the human genome and consists of two main open reading frames (ORFs) encoding the necessary proteins for retrotransposition [2, 3], one is ORF1 encoding a 40 kDa RNA-binding protein, and the other is ORF2 that encodes a 150 kDa protein with endonuclease and reverse transcriptase activities [4].

CpG dinucleotides which are moderately found at LINE-1 promoter, are methylated in the normal cells [5]. Methylation of the LINE-1 promoter and silencing of its transcription is one of the most well-studied mechanisms of LINE-1 repression [6]. The hypomethylation of the LINE-1 promoter increases the accessibility of the RNA polymerase II and the other regulatory elements to initiate or regulate the transcription of this element [7, 8]. It has been shown that in brain cells the young LINE-1 elements that contain truncated or mutated Yin Yang 1 (YY1) binding sites are globally hypomethylated, indicating that the YY1 transcription factor mediates LINE-1 promoter DNA methylation [9]. Binding of Krüppel-associated-box- zinc finger protein (KRAB-ZFP) and recruitment of KAP1 corepressor followed by NURD/HDAC repressor complex, histone methyltransferases, and DNA methyltransferases may as well explain the mechanism of methylation at LINE-1 promoter and several groups of SVA elements [10–12]. The methylation induces silencing of these elements in human ESCs [13]. In addition, it has been shown that PIWI-interacting RNA-induced silencing complex (piRISC) can also guide the de novo methylation machinery to LINE-1 locus [14, 15].

The activity of full-length LINE-1 which can lead to adenoma formation and cancer progression depends on the epigenetic regulation of its promoter [16, 17]. The hypomethylation level at the promoter is very important for activation of LINE-1 elements and by this activation and increasing of their expression, the mobility of these elements is increased, tending to copy and paste around the genome and trying to make new insertions all around the genome [18]. This activity makes the genome more unstable, and also provides a setting for cancer progression [19]. LINE-1 promoter hypomethylation has been observed in various cancers [6]. Several studies have analyzed the LINE-1 hypomethylation in gastrointestinal cancers [20]. Hypomethylation of LINE-1 promoter sequences using methylation-sensitive Southern blotting has detected similar hypomethylated status in normal and colon cancer specimens [21]. However, samples taken from colorectal carcinomas with microsatellite instability have shown a significant decrease in LINE-1 methylation in comparison with normal adjacent tissues using pyrosequencing [22]. Hypomethylation of overall LINE-1 sequence in normal colon mucosa has also been associated with poor survival in patients with sporadic colon cancer [23]. The LINE-1 hypomethylation index (LHI) measured by absolute quantitative analysis of methylated alleles (AQAMA) realtime PCR [24] on paraffin-embedded tissue sections treated by in situ DNA sodium bisulfite modification has shown that LINE-1 is demethylated during the adenomatous and early colorectal cancer (CRC) stages [25]. However, simultaneous analysis of promoter methylation of LINE-1 and quantification of its transcript copy number has not been performed at the adenoma to carcinoma stages of CRC.

In this study, we investigated the relationship between the promoter methylation status and the expression of LINE-1 in colorectal non-advanced adenoma, advanced adenoma, and adenocarcinoma samples. Furthermore, to associate the LINE-1 activation with the mobility and insertion of retrotransposons, we analyzed the insertion of the autonomous LINE-1 and nonautonomous Alu and SVA elements in the advanced adenoma stage (not cancerous) by whole-genome sequencing.

## Methods

### Polyp and cancer biopsies

An informed consent questionnaire briefly describing the research outline was described for, and filled and signed by each patient. Colorectal biopsy samples and polypectomy specimens were acquired from patients at different hospitals and clinics of Mashhad University of Medical Sciences, Mashhad, Iran. The samples were numbered and in cryovials containing RNA Shield (DENAzist Asia Co., Iran) were transferred to liquid Nitrogen within 30 min. Also, the biopsy samples with the same numbers were sent to Mashhad Pathology Laboratory, Mashhad, for histopathological analysis. From each patient, one biopsy sample from the adjacent mucosa was also prepared and processed for deep freezing and histopathological analysis. Portable containers of liquid nitrogen were used to transfer frozen samples to minus eighty freezers at the Research Institute of Biotechnology, Ferdowsi University of Mashhad, for storage and further processing. A total of 5 non-advanced (low-grade) adenomas, 6 advanced (high-grade) adenomas, and 5 cancer (adenocarcinoma) tissue samples along with their adjacent normal mucosa samples were used for analysis of promoter methylation of LINE-1 and quantification of transcript copy numbers. We also performed whole-genome sequencing analysis on six advanced adenomas and one adjacent tissue sample.

### Genomic DNA isolation and sodium bisulfite modification

Genomic DNA was isolated from adenomas, adenocarcinomas, and related adjacent normal tissues using the Animal DNA Isolation Kit (DENAzist Asia Co., Iran). The extracted DNA was subjected to sodium bisulfite modification (SBM). The quality and quantity of extracted DNA and SBM DNA were evaluated using gel electrophoresis and using Epoch 2 nanodrop reader (BioTek Instruments Inc., USA). Bisulfite conversion and subsequent purifications were performed using the EpiTect 96 Bisulfite Kit (Qiagen, GmbH, Hilden, Germany) and according to the manufacturer’s protocols.

### Evaluation of LINE-1 hypomethylation status

**Fig. 1:**
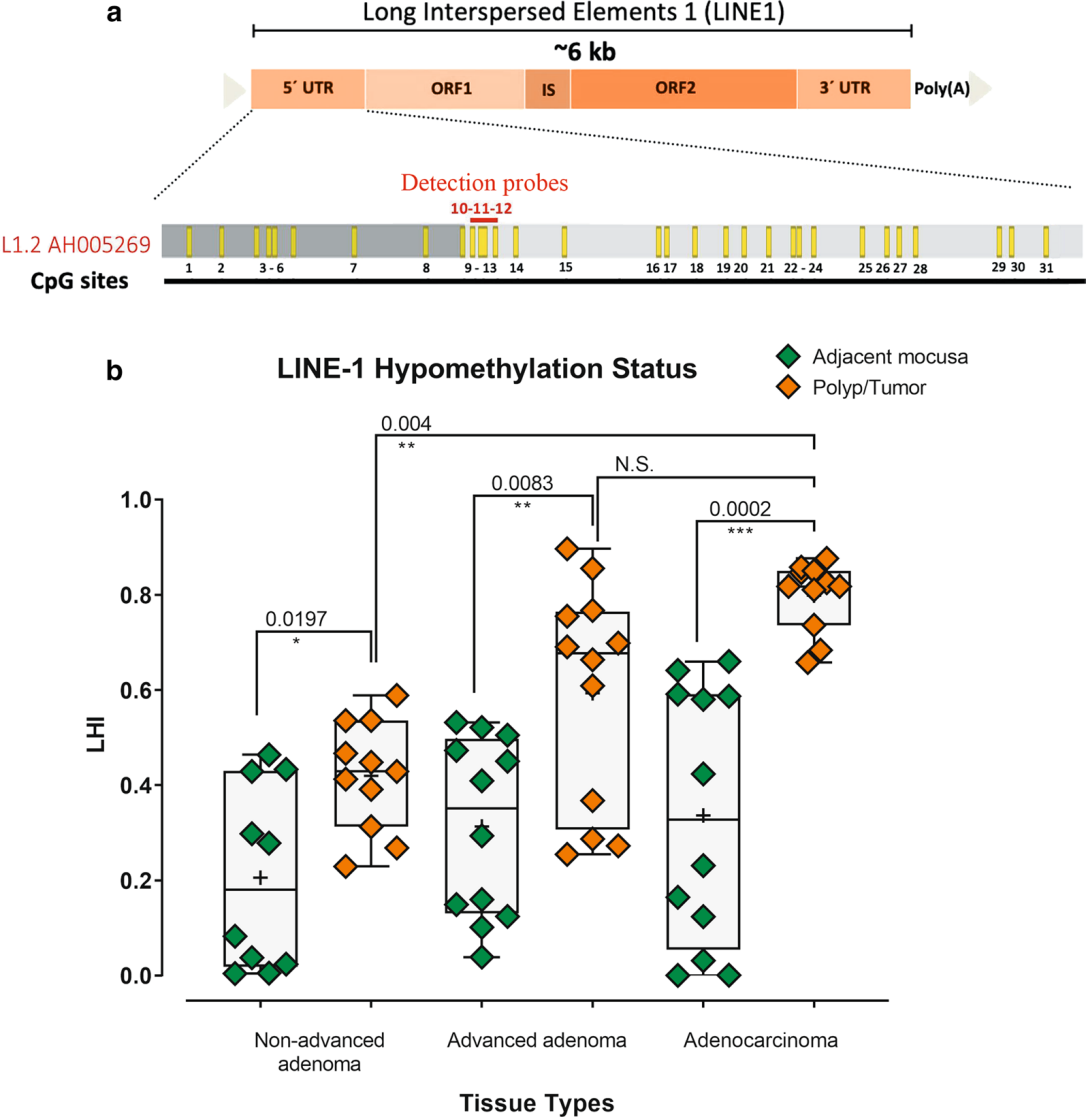
LINE-1 promoter is hypomethylated in colorectal non-advanced adenoma, advanced adenoma, and adenocarcinoma. **a** In the 5′ UTR of LINE-1, there is a cluster of 31 individual CpG sites. The two AQAMA detection probes in this study were able to bind the CpG sites 10, 11, and 12. **b** The univariate scatter/box plot shows the LINE-1 promoter hypomethylation index (LHI) for individual samples in non-advanced adenoma, advanced adenoma, and adenocarcinoma and their adjacent tissues. The Mann–Whitney test was used to analyze the significant differences between different groups of samples at the shown *p*-value. N.S.: not significant

The hypomethylation status of LINE-1 was evaluated by absolute quantitative analysis of methylated alleles (AQAMA) assay [25]. AQAMA requires forward and reverse primers, methylation-specific, and unmethylation-specific TaqMan probes (Table 1; Additional file 1: Figure S1G). A 148-bp fragment at the 5′UTR of the LINE-1 promoter was amplified and subjected to probes to evaluate promoter evaluation. The AQAMA PCR reactions (with a Rotor-Gene Q real-time PCR cycler; Qiagen Inc., USA) were performed in triplicates. Each reaction contained 100 ng of the SBM DNA template, each primer at 500 nM concentration, and dual-labeled hybridization probes (5′FAM-3′BHQ1-labeled for methylation-specific and 5′HEX-3′BHQ1 for unmethylation-specific probe) at 100 nM concentration in Premix Ex Taq (Probe qPCR) master mix (Takara, Japan). The PCR cycles were 95 °C for 5 min, followed by 40 cycles of 94 °C for 30 s, 60 °C for 40 s, and 72 °C for 30 s. QPCR reactions were repeated to adjust the reaction temperature, the concentration of primers, and to acquire the best amplification curves (Additional file 1: Figure S1A–D). In the 5′ UTR of LINE-1, a cluster of 31 individual CpG sites has been identified in a region stretching across approximately 500 bp [26–29]. Our two AQAMA probes were able to bind the CpG sites 10, 11, and 12 (Fig. 1a).

**Table 1:** Oligonucleotides used in this study

Gene	Sequence (5′ To 3′)	Product (bp)	Application
LINE-1 expression primers (GenBank accession number L19088) (25)	F: TGAGAACGGGCAGACAGACT R: AGGTCTGTTGGAATACCCTGCC	129	RT-qPCR
LINE-1 AQAMA Primers	F: GGGTTTATTTTATTAGGGAGTGTTAGA R: TCACCCCTTTCTTTAACTCAAA	148	qPCR
LINE-1 AQAMA methylation specific probe	FAM-TGCGCGAGTCGAAGT-BHQ1	–	qPCR
LINE-1 AQAMA unmethylation specific probe	HEX-TGTGTGAGTTGAAGTAGGG-BHQ1	–	qPCR

To make universal unmethylated and methylated controls, normal peripheral blood DNA was used. Fully unmethylated control (UMC) DNA was synthesized by using Phi-29 DNA polymerase (New England Biolabs) and fully methylated control (MC) was synthesized using M.SssI CpG methyltransferase (New England BioLabs Inc., USA) based on the manufacturer’s protocol and a previous report [30]. All control DNAs were sodium bisulfite converted and were amplified by PCR using primers for LINE-1 promoter. The size of amplified methylated and unmethylated control fragments was confirmed by gel electrophoresis. Then, these fragments were gel-purified and ligated into the pTZ57R_T cloning vector with the TA cloning strategy. The sequence of constructed plasmids containing methylated and unmethylated control fragments was confirmed by Sanger sequencing. Then, the serial dilutions of both constructs were subjected to AQAMA with methylation and unmethylated specific probes to make standard curves. The absolute copy number of each sample was estimated from two standard curves with known copy numbers of methylated and unmethylated DNA (10^1^ to 10^9^ copies) (Additional file 1: Figure S1A and C). The statistical differences for LINE-1 promoter methylation between different groups were tested by the Mann–Whitney test.

### Quantification of LINE-1 transcripts

From non-advanced adenoma, advanced adenoma, and cancer (adenocarcinoma) tissue samples and their adjacent at-risk tissues total RNA was isolated using the Total RNA Isolation Kit (DENAzist Asia Co., Iran). The quality and quantity of extracted RNA were evaluated using gel electrophoresis and spectrophotometry (Epoch 2, BioTek Instruments Inc., USA). One μg of total RNA was reverse transcribed using random hexamer primers and MMLV reverse transcriptase (Thermo Fisher Scientific, USA). To quantify the level of transcripts for LINE-1, quantitative RT-PCR reactions comprising the RealQ Plus 2 × Master Mix Green (containing SYBR Green I; Ampliqon, Denmark), 200 ng cDNA template, and each primer (Table 1) at 500 nM in a 20 μl reaction volume, were carried out in a Rotor-Gene Q real-time PCR cycler (Qiagen, USA). The majority of insertions of LINE-1 are similar to each other in different parts of the genome, and their transcription is directed by “read-through” transcription rather than the LINE-1 promoter [31]. Since we were interested to quantify the LINE-1 RNA that is the intermediate for retrotransposition and is encoded by the LINE-1 promoter, we designed primers that were able to amplify a fragment from cDNA corresponding to the 5′UTR of LINE-1. Amplification steps were 95 °C for 15 min, followed by 40 cycles of 94 °C for 30 s, 57 °C for 30 s, and 72 °C for 30 s. The identity of PCR products was confirmed by melt curve analysis and gel electrophoresis (Additional file 2: Figure S2). To check the genomic DNA contamination, the RT-minus reaction for each sample was included.

QPCR reactions were repeated to adjust the reaction temperature, the concentration of primers, and to acquire the best amplification curves (Additional file 2: Figure S2). Amplified fragments were extracted from agarose gel and after nanodrop spectrophotometry and determination of their serial dilution were used to make quantification standard curves. Each dilution was subjected to triplicate qPCR reactions. The log_10_ of absolute copy numbers was plotted against the relevant cycle threshold (CT) to draw the standard curve. The efficiency of qPCR was calculated from the slope of standard curves, according to the following equation: Efficiency = (10^−1/slope^ − 1) × 100%. The absolute copy numbers for LINE-1 transcripts were quantified based on the standard curve. The statistical differences between differentially expressed groups for the LINE-1 transcript copy number was tested by the Mann–Whitney test.

### Analysis of LINE-1 insertion

Genomic DNA isolated from 6 advanced adenoma tissue samples and 1 adjacent at-risk tissue was prepared for sequencing on the Illumina HiSeq 2500 platform with the High Throughput Library Preparation Kit (Macrogen Inc., South Korea). DNA was fragmented, and the libraries were prepared using a standard version of the manufacturer’s protocol. Libraries were assessed for concentration and fragment size. The libraries were pooled and sequenced on a 100 paired-end Illumina HiSeq 2500 run (Illumina Inc., USA). The samples were sequenced to a depth equivalent to ~ 30X coverage. The resulting sorted BAM files were used for the mobile element indication.

Mobile Element Insertion (MEI) identification was carried out on WGS Illumina data using the MELT (mobile element locator tool Version 2.1.5) algorithm with default parameters (http://melt.igs.umaryland.edu) to find the insertion of different mobile elements like LINE-1, ALU (a primate SINE) and SVA (SINE-VNTR-Alu). MELT detects Alu, LINE-1, and SVA MEIs by searching for signs of split reads (SRs) and discordant read pairs (DRPs) in Illumina WGS data that are enriched at sites containing new and non- reference mobile element insertions. Standard deviation cutoff for the distance between the left and right sides of MEI evidence was 35 and it was used for all MEI detections in the main MELT. The default is appropriate for all standard sequencing library preparations. MELT uses SRs to filter the exact breakpoints and target site duplications at each candidate MEI site. When applied on a wide range, MELT constructs MEI models using all of the available DRPs and SRs data from several samples to precisely discover each MEI site and its features [32]. Different features of insertions including being polymorphic or reference insertions, their size, chromosomal location, incidence, and target regions (3′UTR, 5′UTR, exonic, intronic, intergenic, promoter, transcription terminator) were compared (Additional file 3: Figure S3). Insertion sites were manually examined using Golden Helix Genome Browser software (version 7.8.10). The 1000 genomes version of the hg19 human genome reference sequence (available at ftp://ftp.1000genomes.ebi.ac.uk/vol1/ftp/technical/reference/human_g1k_v37.fasta.gz) was used as a reference sequence to find the new insertions. The circos plots were drawn using ShinyCircos online software (shinycircos.ncpgr.cn). Polymorphic (non-reference) insertions are defined as insertions that do not have conformity with the list of reference MEIs and may be restricted to the related population (hg19).

### Gene ontology analysis

Based on Alu polymorphic (non-reference) insertions in the introns, exons, promoters, terminators, and 3′UTRs of protein-coding genes (PCGs), functional annotation clustering of affected genes with a − log_10_ *p*-value > 2 was performed using the Gene Ontology category (GO Direct) of the latest released version of DAVID web tool (The Database for Annotation, Visualization, and Integrated Discovery v6.8 Oct. 2016) (http://david.ncifcrf.gov). A *p*-value of < 0.01 was considered statistically significant.

## Results

### LINE-1 promoter is hypomethylated in colorectal non-advanced adenoma, advanced adenoma, and adenocarcinoma

To determine the methylation status of LINE-1 promoter in different stages of early colorectal cancer, the LINE-1 Hypomethylation Index (LHI) was analyzed in colorectal non-advanced (low-grade) adenoma, advanced (high-grade) adenoma, and adenocarcinoma and in their adjacent at-risk (control) tissues. In all three stages, the polyp/tumor tissue samples had significantly lower methylation levels (higher LHI) than their control adjacent counterparts (Fig. 1). Also, the methylation levels of LINE-1 promoter in advanced adenoma and adenocarcinoma samples were significantly lower than those in non-advanced adenomas. The finding of LINE-1 promoter hypomethylation in non-advanced adenomas (low-grade polyps) seems to be one of the earliest events for tumor formation (Fig. 1).

### Increased LINE-1 transcripts in colorectal adenoma, advanced adenoma, and adenocarcinoma

**Fig. 2:**
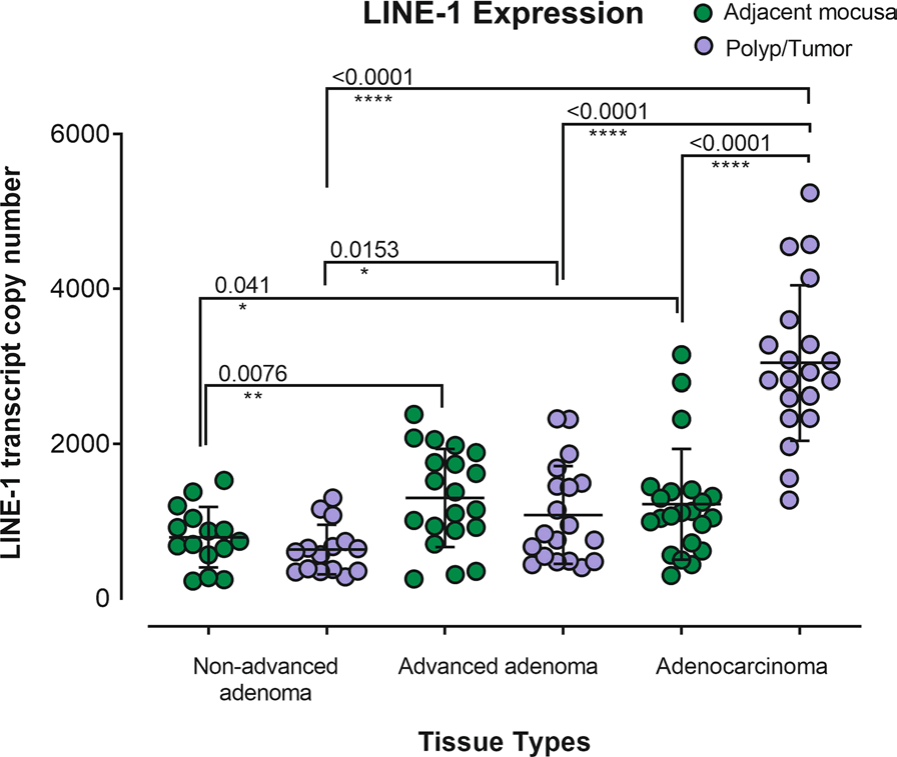
Increased LINE-1 transcripts in colorectal adenoma, advanced adenoma, and adenocarcinoma. The univariate scatter plot shows the differential expression of LINE-1 transcript for individual samples in non-advanced adenoma, advanced adenoma, and adenocarcinoma and their adjacent tissues. The Mann–Whitney test was used to analyze the significant differences between different groups of samples at the shown *p*-value

The progressively increased LINE-1 promoter hypomethylation from non-advanced adenoma to advanced adenoma and adenocarcinoma suggested that there could be an association between the progressing levels of promoter hypomethylation and transcriptional activation of LINE-1 during the development of colorectal cancer. RT-qPCR quantification of LINE-1 transcripts revealed that the LINE-1 transcripts in advanced adenoma were significantly higher than those in non-advanced adenomas, and in the adenocarcinoma, they were significantly higher than those in the advanced adenoma (Fig. 2). The LINE-1 transcript levels in adenocarcinomas were significantly higher than those in the adjacent tissues. This significant level of increased transcripts between the polyp and adjacent tissues was not observed in non-advanced and advanced adenomas, while the overall level of LINE-1 transcripts was higher in advanced adenoma and the adjacent tissues than that in non-advanced adenoma and the relevant adjacent tissues (Fig. 2).

### Somatic insertion of mobile elements in the genome of colorectal advanced adenomas

**Fig. 3:**
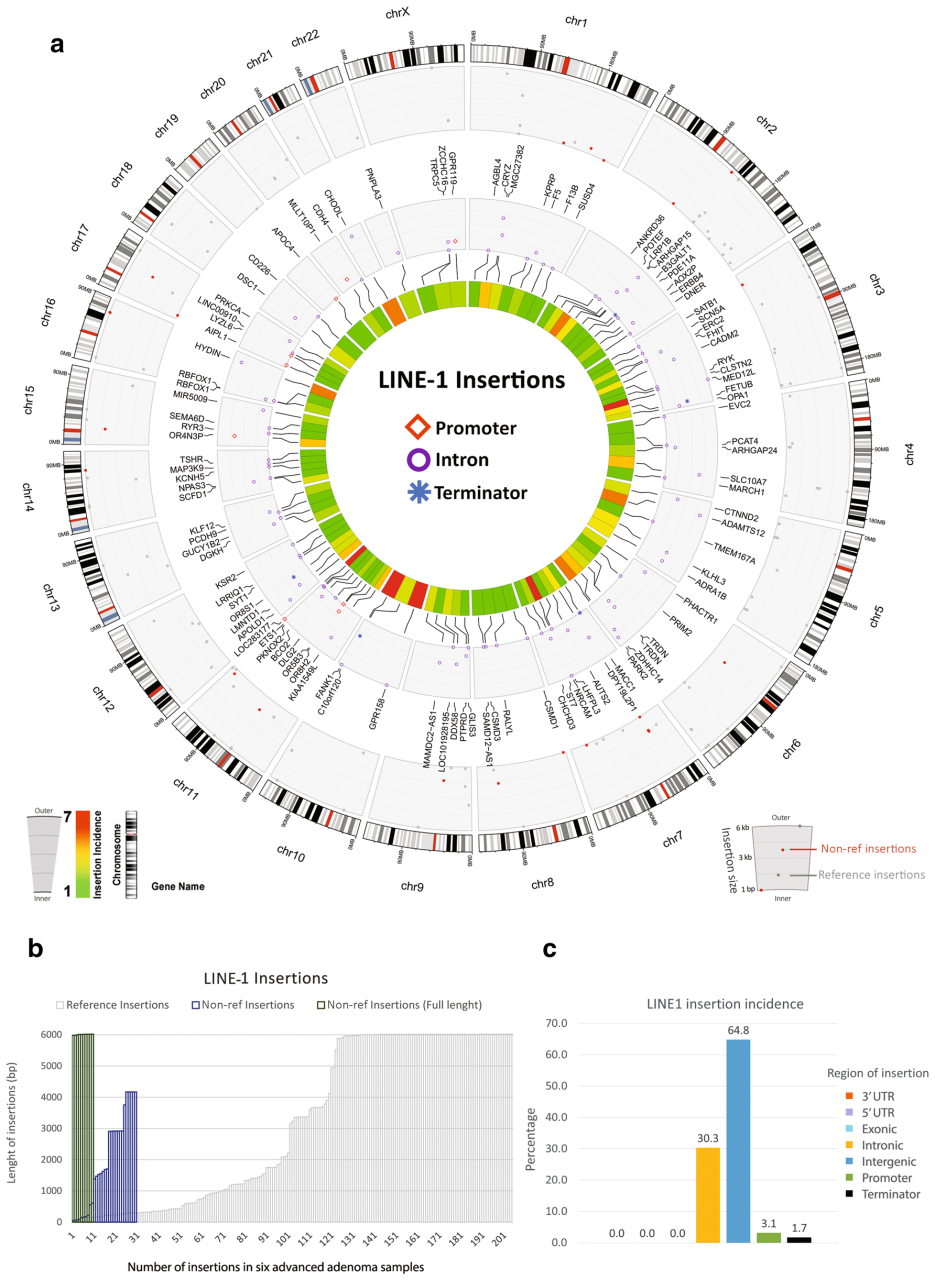
Somatic insertion of LINE-1 elements in the genome of colorectal advanced adenomas. **a** The circos plot shows the incidence and location of LINE-1 insertions in six advanced adenoma polyp tissue compared to one normal mucosa. The outer track shows the location of insertions in relation to cytobands of chromosomes. From outside to inside, the second track shows the size and type of insertions (non-reference or reference) (the right guide inset). From outside to inside, the third track shows the genes targeted by insertions and target regions (promoters, introns, and transcription termination regions). The innermost heatmap track shows the incidence of insertion for each genomic region (see the left guide inset). **b** Length and number of LINE-1 reference, non-reference, and non-reference full-length insertions. **c** Percentage of LINE-1 insertions in each of the genomic regions

The advanced adenoma stage is a critical and transient state between the initial polyps (adenomas) and adenocarcinomas. Thus, we decided to analyze the genome of advanced adenomatous tissues to identify the location, size, incidence, and types of mobile elements. For this purpose, MELT analysis was performed on the whole genome sequencing data compared to the reference genome (1000 Genomes Project MEIs). MELT using Burroughs-Wheeler Alignment to align all reads properly to the reference genome and reference insertion list, allowed us to find and compare non-reference insertions. Each new LINE-1 insertion has specific characteristics of retrotransposition including random 5′ inversion, numerous 5′ truncation, a poly(A) tail, and flanking target site duplications (TSDs) [33]. In comparison with six advanced adenoma samples and one adjacent tissue, and based on the size, insertions in different genomic locations were identified (Fig. 3a). The ratio was approximately 16% acquired by dividing polymorphic (non-reference) insertions to the total of LINE-1 insertions in advanced adenoma samples (Fig. 3b; Table 2). One-third of these insertions comprised full length (~ 6 kb) elements (Fig. 3b). The highest to lowest percentage of LINE-1 insertions were observed in intergenic (64.8%), intronic (30.3%), promoter sequences (3.1%), and transcription terminator regions (1.7%) (Fig. 3c).

**Table 2:** Non-reference LINE-1 insertions in six colorectal advanced adenomas and one adjacent tissue

NCBI-ID	Gene name	Gene full name	Region of insertion	Insertion family Type	Size of Insertion (bp)	Sample Count
NR_028067	OR4N3P	olfactory receptor family 4 subfamily N member 3 pseudogene	PROMOTER	L1Ambig	4170	5
NM_001005469	OR5B3	olfactory receptor family 5 subfamily B member 3	PROMOTER	L1Ta	1471	1
NM_001033054	AIPL1	aryl hydrocarbon receptor interacting protein like 1	PROMOTER	L1Ambig	64	1
NM_001270974	HYDIN	HYDIN, Axonemal Central Pair Apparatus Protein	INTRONIC	L1Ta1d	*5984–6019	6
NM_000369	TSHR	Thyroid Stimulating Hormone Receptor	INTRONIC	L1Ta1d	*6017	1
NM_001099771	POTEF	POTE Ankyrin Domain Family Member F	INTRONIC	L1Ta1d	*5986–6010	3
NR_027413	LINC00910	Long Intergenic Non-Protein Coding RNA 910	INTRONIC	L1Ambig	3756	1
NM_145235	FANK1	Fibronectin Type III And Ankyrin Repeat Domains 1	INTRONIC	L1Ambig	2910–2919	7
NM_052900	CSMD3	CUB and Sushi Multiple Domains 3	INTRONIC	L1Ambig	1705	1
NR_002833	DPY19L2P1	DPY19L2 Pseudogene 1	INTRONIC	L1Ambig	83–1683	4
NM_000947	PRIM2	Primase DNA Subunit 2	INTRONIC	L1Ambig	1525	1
NR_135597.1	LOC101928195	–	INTRONIC	L1Ambig	1386	1
NM_001994	F13B	Coagulation Factor XIII B Chain	INTRONIC	L1Ta	604	1
NM_022062	PKNOX2	PBX/Knotted 1 Homeobox 2	INTRONIC	L1Ambig	547	1
NM_001025231	KPRP	Keratinocyte Proline Rich Protein	INTRONIC	L1Ta	218	1
NM_001164315.1	ANKRD36	Ankyrin Repeat Domain 36	INTRONIC	L1Ambig	179	1
NM_001131010	SATB1	SATB Homeobox 1	INTRONIC	L1Ambig	159	1
NM_017812	CHCHD3	Coiled-Coil-Helix-Coiled-Coil-Helix Domain Containing 3	INTRONIC	L1Ambig	147	1
NM_001037175	SUSD4	Sushi Domain Containing 4	INTRONIC	L1Ambig	59	1
NM_033225	CSMD1	CUB And Sushi Multiple Domains 1	INTRONIC	L1Ambig	49	1

* Full-length insertion event

**Fig. 4:**
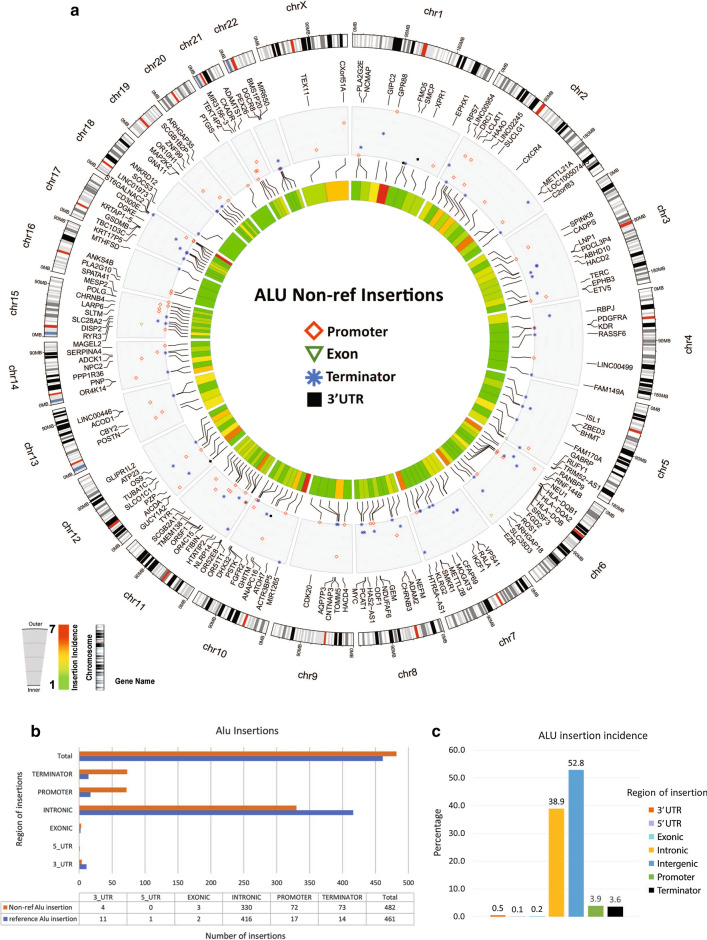
Somatic non-reference insertion of Alu elements in the genome of colorectal advanced adenomas. **a** The circos plot shows the incidence and location of Alu insertions in six advanced adenoma polyp tissue compared to one normal mucosa. The outer track shows the location of insertions in relation to cytobands of chromosomes. From outside to inside, the second track shows the genes targeted by insertions and target regions (promoters, exons, transcription termination regions, and 3′ UTR). The innermost heatmap track shows the incidence of insertion for each genomic region (see the left guide inset). **b** The region and number of Alu reference and non-reference insertions. **c** Percentage of Alu insertions in each of the genomic regions

**Fig. 5:**
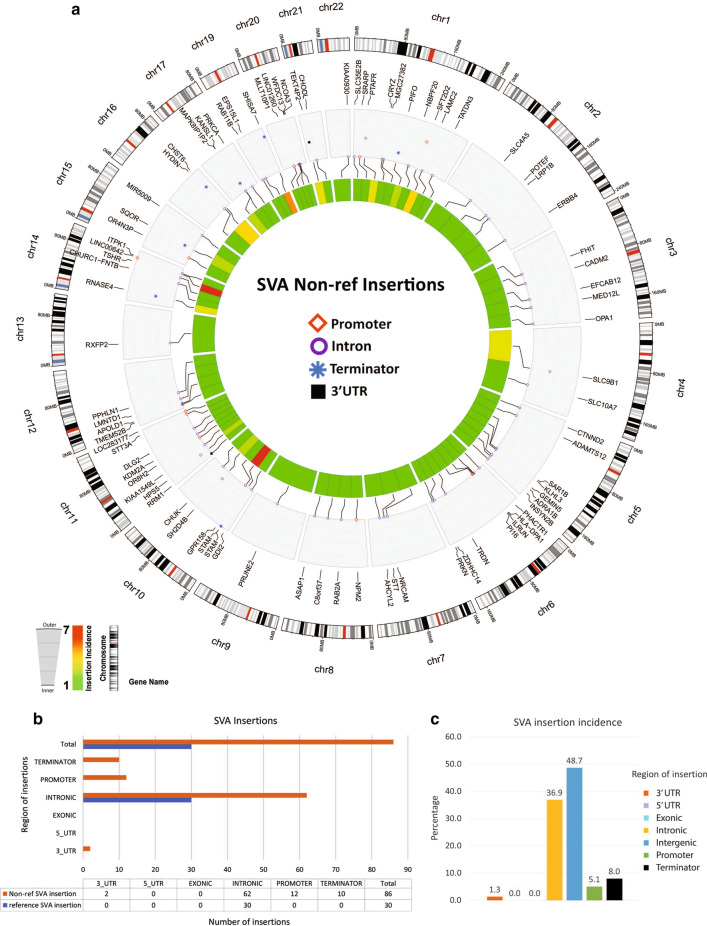
Somatic non-reference insertion of SVA elements in the genome of colorectal advanced adenomas. **a** The circos plot shows the incidence and location of SVA insertions in six advanced adenoma polyp tissue compared to one normal mucosa. The outer track shows the location of insertions in relation to cytobands of chromosomes. From outside to inside, the second track shows the genes targeted by insertions and target regions (promoters, introns, transcription termination regions, and 3′ UTR). The innermost heatmap track shows the incidence of insertion for each genomic region (see the left guide inset). **b** The region and number of SVA reference and non-reference insertions. **c** Percentage of SVA insertions in each of the genomic regions

Alu and SVA elements remain active and are mobile in the genome. We found that similar to LINE-1, the highest and the second-highest incidence of Alu and SVA insertions occurred in intergenic and intronic regions (Figs. 4 and 5). In addition, the number of insertions for Alu elements in colorectal advanced adenoma tissues was more than those for LINE-1 and SVA elements. Also, non-reference insertions of Alu were markedly higher than those for LINE-1 and SVA elements, and Alu was the only element that showed polymorphic insertion in the exonic regions (Fig. 4b). Three genes that were targeted by Alu in their exons were RUFY1, EZR, and RYR3 in chromosomes 5, 6, and 15, respectively (Fig. 4a). While SVA elements had the lowest number of insertions in comparison with LINE-1 and Alu elements, they had the highest percentage of insertions in the transcription termination regions (Fig. 5b, c).

### LINE-1 and Alu insertions within the protein-coding genes

**Fig. 6:**
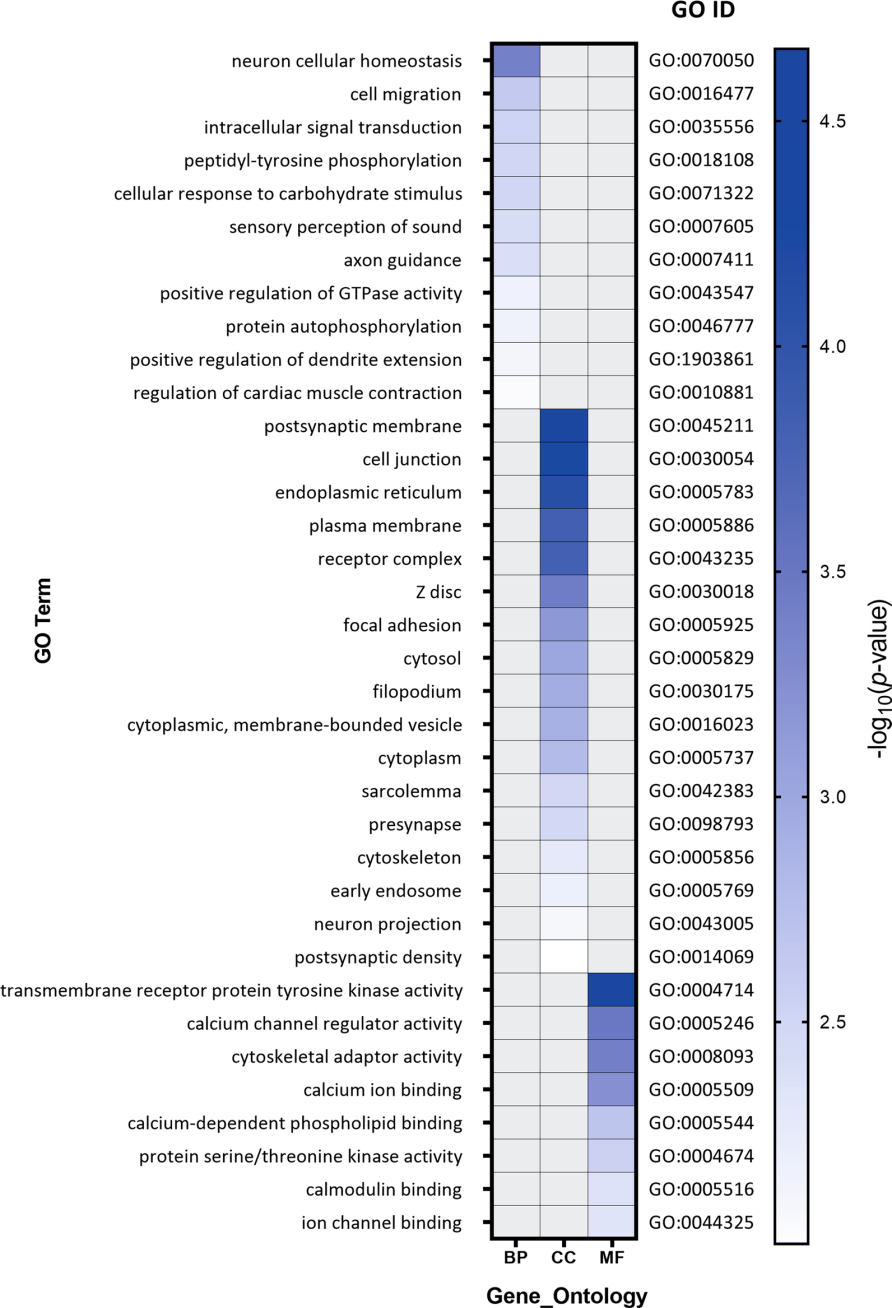
Heatmap of gene ontology (GO) term enrichment analysis of genes that are affected by Alu retrotransposition in advanced adenomas. GO terms indicate genes that are affected by Alu non-reference insertions in three categories of biological processes (BP), cellular component (CC), and molecular function (MF). Lower (more significant) *p*-values indicating enrichment for the term are colored in blue. The higher *p*-values are indicated in shades of blue toward white

**Fig. 7:**
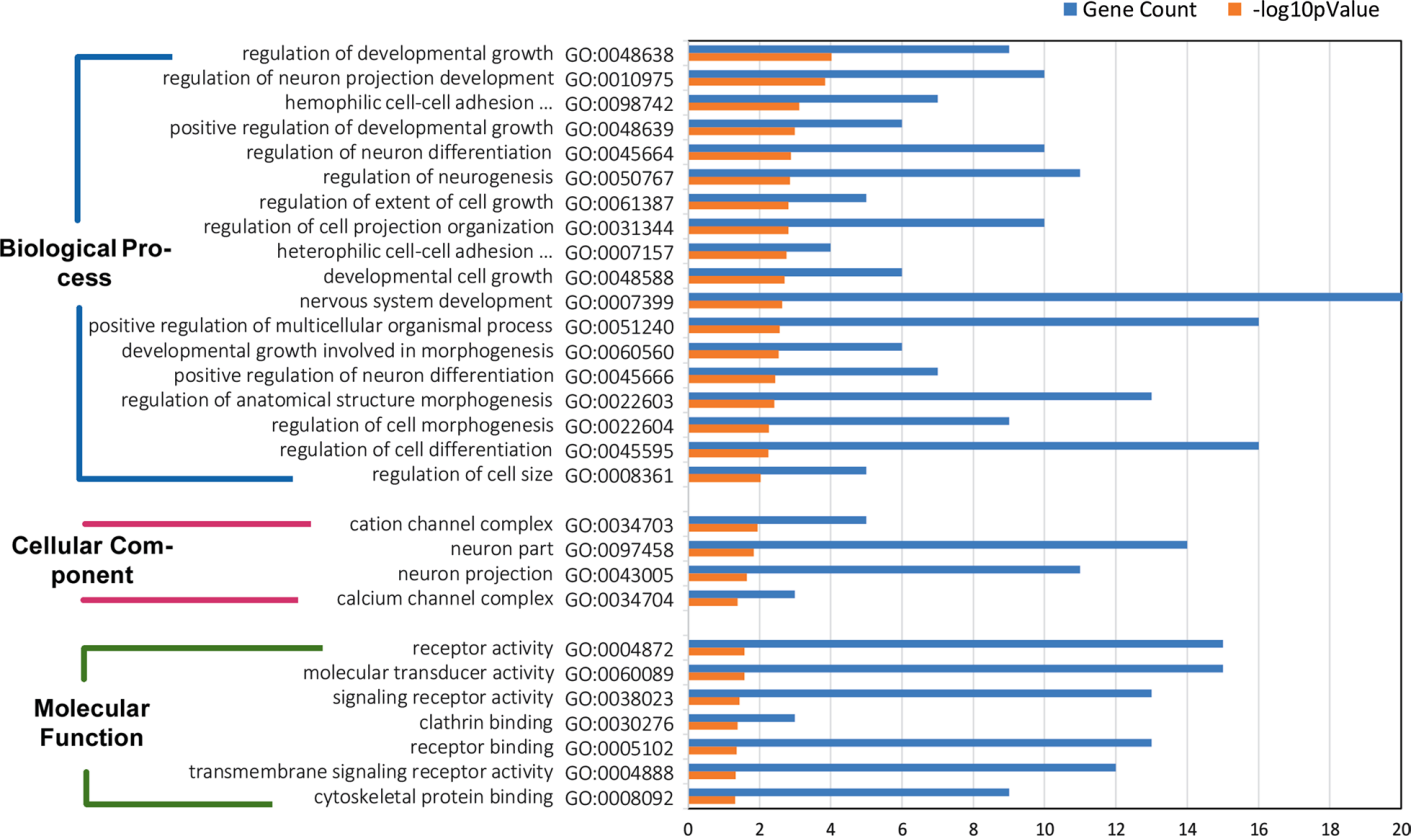
Gene ontology analysis of advanced adenomas affected by LINE-1 retrotransposition. For each category of biological processes, cellular components, and molecular functions, those terms that were significantly associated with the gene list were plotted with the gene count and the −log_10_p value. The counted genes are those that had LINE-1 insertions in their intronic, promoter, and transcription terminator regions

Based on the enrichment analysis of LINE-1 and Alu polymorphic insertions in the affected gene, the GO terms in three categories of molecular function (MF), biological process (BP), and cellular components (CC) were determined (Figs. 6 and 7). The most important BPs based on the *p*-value for Alu polymorphic insertions were neuron cellular homeostasis and cell migration. Postsynaptic membrane and cell junction were the most important CCs that were affected by Alu insertions. Transmembrane receptor protein tyrosine kinase activity and calcium channel regulator activity were molecular functions that were significantly affected by Alu polymorphic insertions (Fig. 6). For LINE-1 insertions in the BP category, the most important term based on gene enrichment was regulation of developmental growth, and one of the most important terms based on gene count was regulation of cell differentiation. In the MF category, the most important terms affected by the LINE-1 insertions based on gene enrichment and gene count were receptor activity and molecular transducer activity (Fig. 7).

## Discussion

In this study, we investigated the relationship between the promoter methylation and the expression of LINE-1 in three stages of colorectal non-advanced adenoma, advanced adenoma, and adenocarcinoma. In addition, we analyzed the insertion of LINE-1, Alu, and SVA elements in the genome of colorectal advanced adenomas. We found that in all three stages, the polyp/tumor tissue had significantly lower methylated CpG dinucleotides in the LINE-1 promoter than their adjacent control tissues (Fig. 1). Also, the LHI in advanced adenoma and adenocarcinoma were significantly higher than those in non-advanced adenomas (Fig. 1). The copy number of LINE-1 transcripts in advanced adenoma was significantly higher than that in non-advanced adenomas, and in adenocarcinomas was significantly higher than that in the advanced adenomas (Fig. 2). Analysis of the genome of colorectal advanced adenomas revealed that at this stage polymorphic insertions of LINE-1, Alu, and SVA were approximately 16%, 51%, and 74%, respectively (Figs. 3, 4, 5).

Numerous cytoplasmic and nuclear mechanisms can restrict or inhibit non-LTR retrotransposon activity and mobilization. These restriction mechanisms are reviewed by Goodier [34]. Regulation of methylation of CpG islands is one of the most important restriction mechanisms that is controlled by several interacting factors to restrict or inhibit LINE-1 activity. In human cells, these factors include DNA methyltransferases (such as DNMT1, DNMT3α, and DNMT 3β), DNA methyl-binding proteins (such as methyl CpG binding protein 2), histone methyltransferases (such as SUV39H), DNA repair proteins such as ATM serine/threonine kinase and ERCC excision repair 4, KRAB-ZFPs and their corepressors (such as PLZF, ZNF91, ZNF93, and KAP1), and transcription factors (such as YY1, P53, RUNX3, SP1, SOX2, RAR, and ETS1) [13, 35–44]. The exact mechanisms to explain the escape of retroelements from the inhibitory mechanisms of cells are unknown. For example, the loss of methylation marks at CpG islands in cancer cells could be secondary to a genome-wide hypomethylation event, or a concerted function of effector enzymes, transcription factors, long noncoding RNAs and regulatory proteins working on a specific promoter [45]. The loss or gain of interaction of long noncoding RNAs with DNA methyltransferases could be very important for LINE-1 expression. For instance, studies have shown that CCAT1 long noncoding RNA which is involved in the regulation of proliferation and anchorage-independent growth of cancer cells and affects other regulatory lncRNAs may function as a scaffold for epigenetic regulators of gene function and determine CpG island methylator phenotype [46–49]. One of our observations was that the normal (at risk) mucosa adjacent to advanced and adenocarcinoma had a significantly higher copy number of LINE-1 transcripts than the normal mucosa adjacent to non-advanced adenomas (Fig. 2). This indicates that LINE-1 expression changes are detectable in the adjacent normal mucosa, even at levels similar to the polyp tissue. The increased expression of LINE-1 is dramatically elevated in the adenocarcinoma tissue (Fig. 2). These observations are in line with the results of a previous study indicating that the genomic profiles of at-risk mucosa and adenomas demonstrate the evolution from normal tissue to carcinoma, and substantial genomic variations exist in at-risk mucosa even before adenoma formation [50]. Another study has suggested the presence of a field-effect affecting both adjacent and non-adjacent normal mucosa in CRC [51]. If we extend these field effects to epigenetic alterations [52], it is conceivable that the altered expression of the adjacent (at-risk) mucosa may involve LINE-1 transcription.

An interesting aspect of the LINE-1 function is its relation to the pluripotency status of the cell. It has been shown that its transcriptional activation regulates chromatin accessibility in the early mouse embryo, and it is involved in nuclear organization and cellular identity of embryonic stem cells [53, 54]. Thus, the activity of LINE-1 may also be secondary to pluripotency and cancer stemness, where transcription factors and methyltransferases cooperate to establish uncontrolled proliferation, enhanced self-renewal, increased migration, and elevation of differentiation potential into different cell types [55, 56].

Another aspect of LINE-1 function and biology that emphasizes the importance of LINE-1 expression studies relates to the retrotransposition of other elements in the genome. Alu and SVA are the non-autonomous non-LTR retrotransposons that can hijack LINE-1 retrotransposon machinery, to use the LINE-1-encoded proteins for their mobilization [33, 57]. It has been suggested that endogenous ORF1 and ORF2 of LINE-1 could assist to retrotranspose Alu elements, and SVA is the preferred substrate for the L1-encoded protein machinery [33, 58–60]. Alu elements are the most successful transposons in the human genome which are transcribed by RNA polymerase III [57]. Studies suggest that the retrotransposition rate of Alu is ten times higher than LINE-1 [61]. We found that the number of total and polymorphic insertions for Alu elements in colorectal advanced adenoma tissues were more than those for LINE-1 and SVA elements. Alu was the only element that showed polymorphic insertions in the exonic regions (Figs. 4 and 6). Three genes that were targeted by Alu in their exons were RUFY1, EZR, and RYR3. RUN And FYVE Domain Containing 1 (RUFY1) gene encodes a protein that contains a RUN domain and a FYVE-type zinc finger domain, playing a role in early endosomal trafficking. A recent study has reported the silencing of RUFY1 expression in gastric cancer cells [62]. EZR (Ezrin) protein functions as a protein-tyrosine kinase substrate in microvilli and plays a key role in cell surface structure adhesion and migration. Several studies have reported the importance of this molecule in colorectal cancer [63–65]. Ryanodine Receptor 3 (RYR3) is involved in calcium release from intracellular storage. These receptors have also been reported as important players in colorectal cancer [66, 67]. In metastatic colorectal cancer, the inadvertent activation of evolutionarily methylation-silenced genes MET, RAB3IP and CHRM3 proto-oncogenes have been identified [68].

Previous studies have tried to identify hotspots for LINE-1 insertion. A study on Hela cells found that there are three hotspots for LINE-1 insertion in chromosomes 1, 5, and 12 [69]. Another study on 202 colorectal cancers has reported that 15 genes from 291 cancer census genes [70] display recurrent insertions [71]. Three of these genes, LDL receptor related protein 1B (LRP1B), Erb-B2 receptor tyrosine kinase 4 (ERBB4), and fragile histidine triad diadenosine triphosphatase (FHIT), are the targets of retrotransposition identified in our study (Figs. 3, 4, 5). Another gene reported by the study on CRC samples was discs large MAGUK scaffold protein 2 (DLG2) which is frequently targeted by retrotransposition [71]. It is interesting that in this study on advanced adenoma samples, we have found retrotransposition of both LINE-1 and SVA elements in all these four genes (Figs. 3 and 5). Three genes of LRP1B, FHIT, and DLG2 belong to a group of 9 genes that have been reported to be the most frequently integrated spots for HPV in cervical carcinomas [72].

Besides the insertional mutagenesis caused by the mobilization of retroelements and the genes that lose their encoding ability, other mechanisms for carcinogenesis of these elements have been postulated. The antisense promoter of human LINE-1 can start the transcription of adjacent genomic sequences generating chimeric RNAs that can perturb transcription of neighboring genes. These chimeric transcripts that have been isolated from breast cancer cell lines and primary tumors and colon cancer cells seem to be unique and of biomarker value for the determination of malignancy [73]. Indeed, the location of insertions being at the promoter, 5′ UTR, exons, introns, 3′ UTR, and transcription termination sequences would be a determining factor. For example, intronic LINE-1 insertion into a regulatory element has increased the expression of the candidate liver oncogene ST18 [74]. Studies have also confirmed that intronic LINE-1 insertions result in decreased expression of the mutated genes [6, 75]. This could theoretically contribute to tumorigenesis by decreasing the expression of tumor suppressor genes. We found that the highest and the second-highest incidence of LINE-1, Alu, and SVA insertions occurred in intergenic and intronic regions (Figs. 3, 4, 5). SVA had the highest percentage of insertions in the transcription termination regions. All three elements showed 3.9–5.1% insertions in promoters. A recent genome-wide study on somatic insertions in 201 samples shows that retrotranspositions are predicted to initiate approximately 1% of CRCs [71]. This study reports that tumors with high retrotransposon activity demonstrate features of MSS and MSI tumors, and insertion count is independently associated with poor disease-specific survival [71].

## Conclusions

In summary, we have shown that methylation of CpG islands in the LINE-1 promoter is progressively decreased in each of the three stages of non-advanced adenoma, advanced adenoma, and adenocarcinoma. This stage-to-stage decreased methylation which overlaps onto the course of colorectal cancer was accompanied by a higher level of LINE-1 transcription, suggesting that retrotransposition of LINE-1 and non-autonomous retroelements might be driving cancer. Polymorphic insertions which were found in advanced (high-grade) adenoma, a precancerous stage before colorectal carcinoma, leads us to speculate that the early and advanced polyp stages may host very important pathogenic processes concluding to cancer.

## Supplementary information

**Additional file 1: Figure S1.** Amplification and standard curves and schematic for AQAMA assay.

**Additional file 2: Figure S2.** Standard, amplification, and melting curves for quantification of LINE-1 transcripts.

**Additional file 3: Figure S3.** Representative insertion features for one example of the full length insertion of LIINE-1.

## Data Availability

The data sets used and/or analyzed during the current study are available from the corresponding author on reasonable request.

## Abbreviations

SVA: SINE-R-VNTR-Alus
ORF: Open reading frame
YY1: Yin Yang 1
KRAB-ZFP: Krüppel-associated-box-zinc finger protein
piRISC: PIWI-interacting RNA-induced silencing complex
LHI: LINE-1 hypomethylation index
TSDs: Target site duplications
MF: Molecular function
BP: Biological process
CC: Cellular component
DNMT: DNA methyltransferase
AQAMA: Absolute quantitative analysis of methylated alleles
MEI: Mobile element insertion
