# Supplementary figures and images for: Promoter methylation, transcription, and retrotransposition of LINE-1 in colorectal adenomas and adenocarcinomas
Source: Cancer Cell Int. 2020 Sep 1;20:426. doi: 10.1186/s12935-020-01511-5 (PMC7466817; doi:10.1186/s12935-020-01511-5)

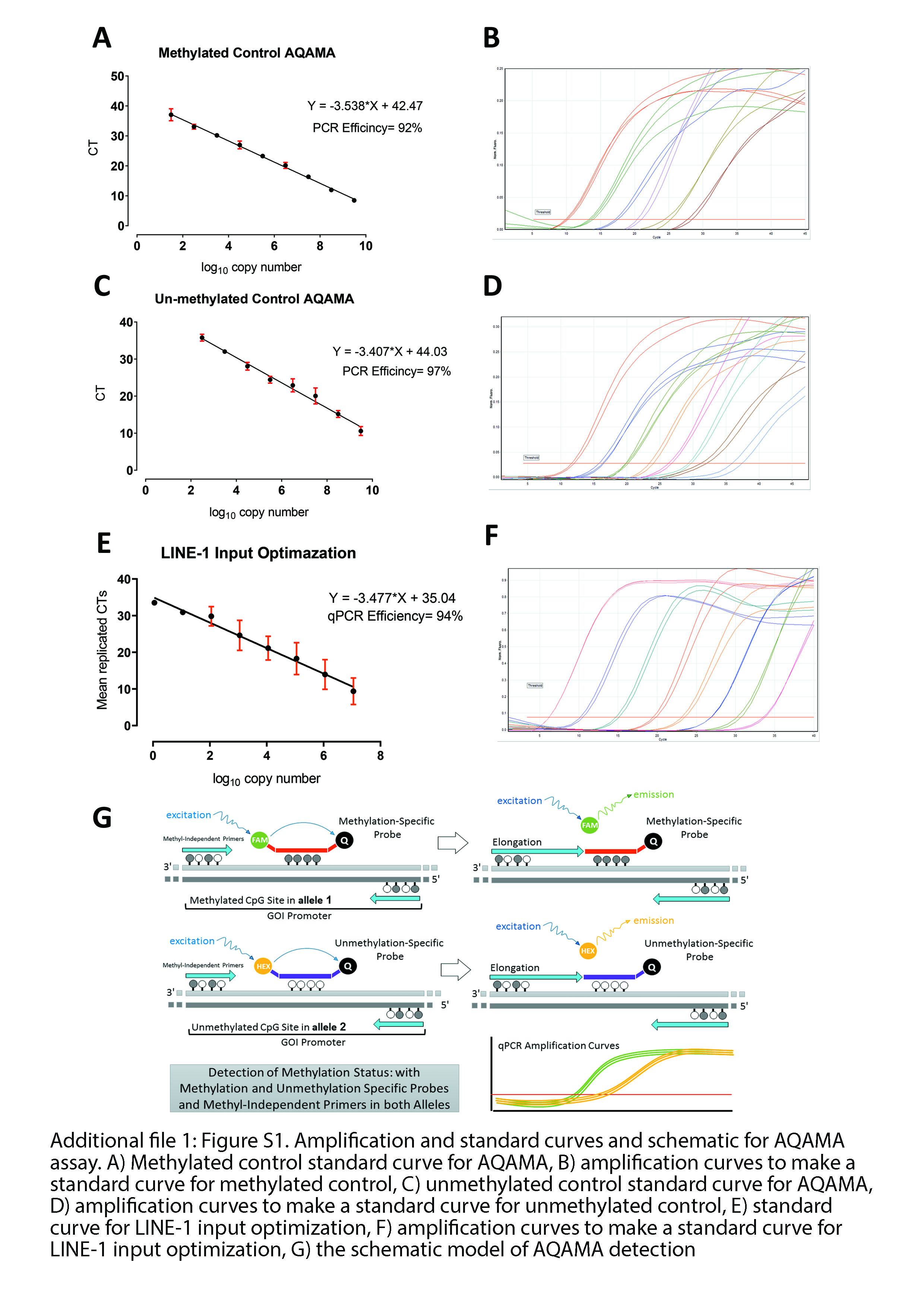

Supplement: Supplementary file 1 — Additional file 1: Figure S1. Amplification and standard curves and schematic for AQAMA assay. [file 12935_2020_1511_MOESM1_ESM.tif]

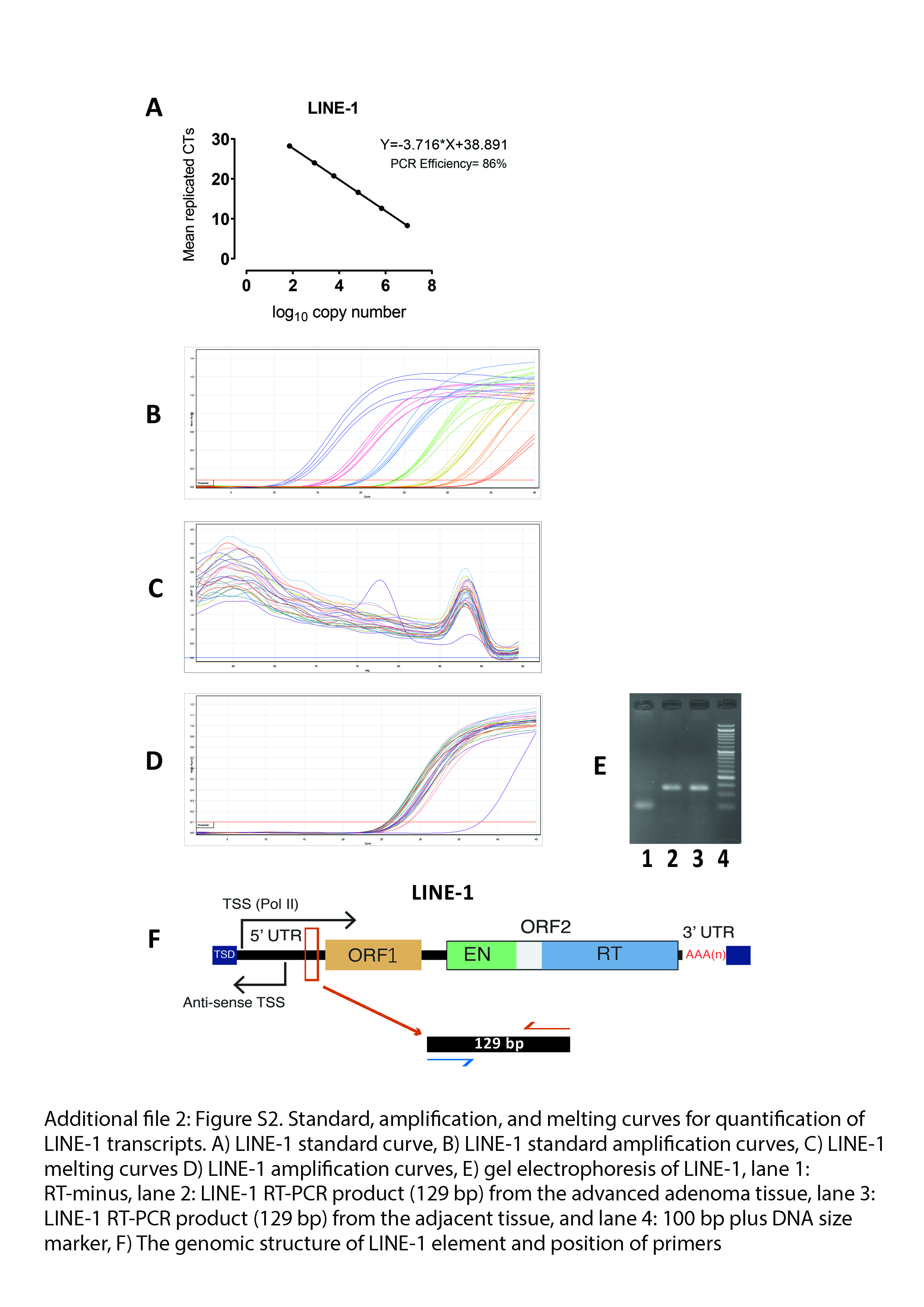

Supplement: Supplementary file 2 — Additional file 2: Figure S2. Standard, amplification, and melting curves for quantification of LINE-1 transcripts. [file 12935_2020_1511_MOESM2_ESM.tif]

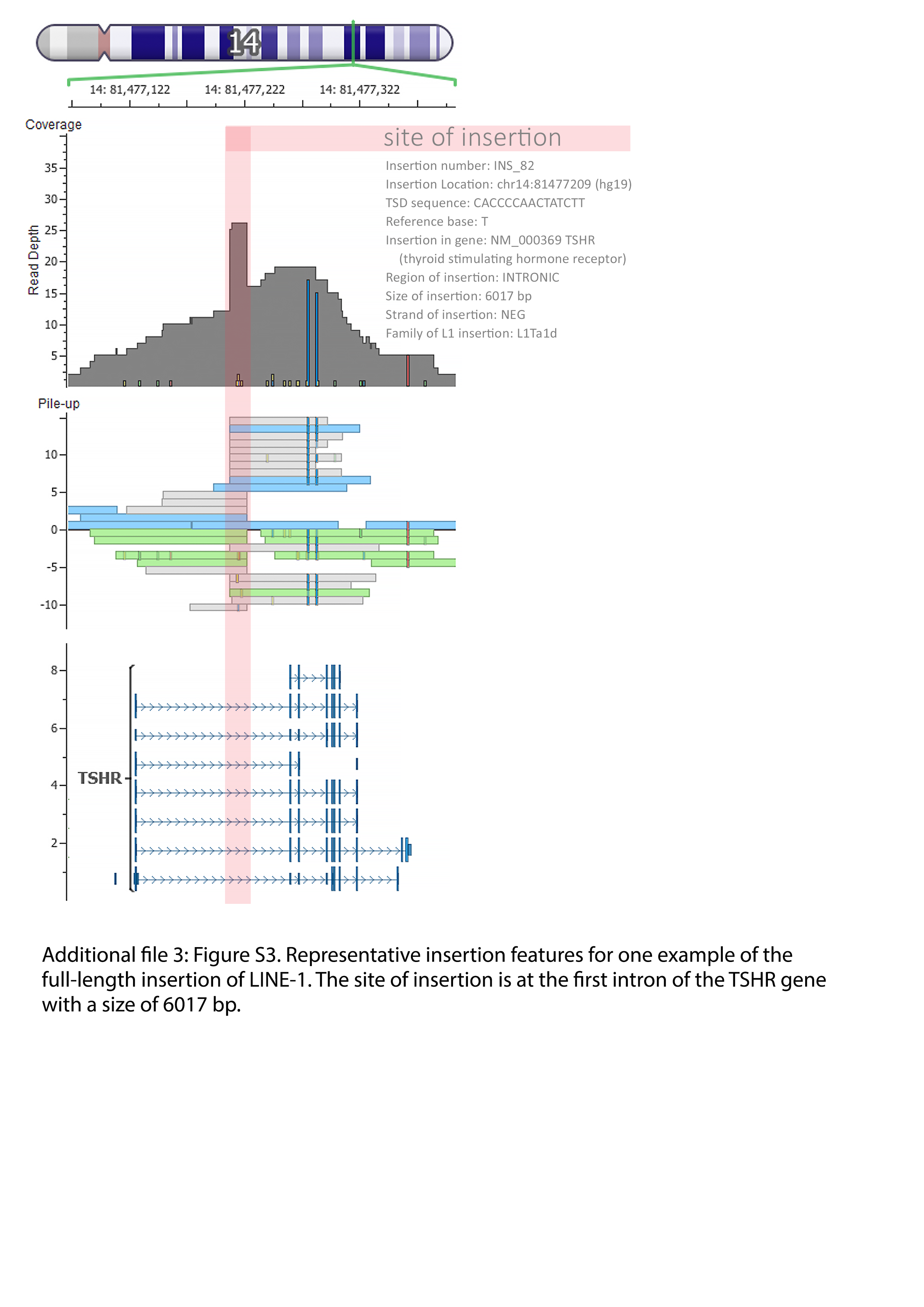

Supplement: Supplementary file 3 — Additional file 3: Figure S3. Representative insertion features for one example of the full length insertion of LIINE-1. [file 12935_2020_1511_MOESM3_ESM.tif]
